# PKA and HOG signaling contribute separable roles to anaerobic xylose fermentation in yeast engineered for biofuel production

**DOI:** 10.1371/journal.pone.0212389

**Published:** 2019-05-21

**Authors:** Ellen R. Wagner, Kevin S. Myers, Nicholas M. Riley, Joshua J. Coon, Audrey P. Gasch

**Affiliations:** 1 Great Lakes Bioenergy Research Center, University of Wisconsin–Madison, Madison, WI United States of America; 2 Department of Chemistry, University of Wisconsin–Madison, Madison, WI United States of America; 3 Genome Center of Wisconsin, University of Wisconsin–Madison, Madison, WI United States of America; 4 Department of Biomolecular Chemistry, University of Wisconsin–Madison, Madison WI United States of America; 5 Morgridge Institute for Research, Madison, WI United States of America; 6 Laboratory of Genetics, University of Wisconsin–Madison, Madison, WI United States of America; Texas A&M University College Station, UNITED STATES

## Abstract

Lignocellulosic biomass offers a sustainable source for biofuel production that does not compete with food-based cropping systems. Importantly, two critical bottlenecks prevent economic adoption: many industrially relevant microorganisms cannot ferment pentose sugars prevalent in lignocellulosic medium, leaving a significant amount of carbon unutilized. Furthermore, chemical biomass pretreatment required to release fermentable sugars generates a variety of toxins, which inhibit microbial growth and metabolism, specifically limiting pentose utilization in engineered strains. Here we dissected genetic determinants of anaerobic xylose fermentation and stress tolerance in chemically pretreated corn stover biomass, called hydrolysate. We previously revealed that loss-of-function mutations in the stress-responsive MAP kinase *HOG1* and negative regulator of the RAS/Protein Kinase A (PKA) pathway, *IRA2*, enhances anaerobic xylose fermentation. However, these mutations likely reduce cells’ ability to tolerate the toxins present in lignocellulosic hydrolysate, making the strain especially vulnerable to it. We tested the contributions of Hog1 and PKA signaling via IRA2 or PKA negative regulatory subunit BCY1 to metabolism, growth, and stress tolerance in corn stover hydrolysate and laboratory medium with mixed sugars. We found mutations causing upregulated PKA activity increase growth rate and glucose consumption in various media but do not have a specific impact on xylose fermentation. In contrast, mutation of *HOG1* specifically increased xylose usage. We hypothesized improving stress tolerance would enhance the rate of xylose consumption in hydrolysate. Surprisingly, increasing stress tolerance did not augment xylose fermentation in lignocellulosic medium in this strain background, suggesting other mechanisms besides cellular stress limit this strain’s ability for anaerobic xylose fermentation in hydrolysate.

## Introduction

Lignocellulosic biomass offers a sustainable source for bioenergy. The use of leftover agriculture byproducts and plants grown on marginal lands for biofuel production reduces waste and removes dependency on food-based cropping systems. Notably, there are two major bottlenecks for sustainable biofuel production from lignocellulosic material. First, many microbes, including industrially relevant *Saccharomyces cerevisiae*, cannot innately ferment pentose sugars like xylose, which comprise a significant fraction of the sugars released from deconstructed biomass [1]. Second, the harsh chemical treatment of plant biomass required to release lignocellulosic sugars produces a variety of toxins and stresses that inhibit microbial growth and fermentation [2,3]. A goal for the biofuel industry is to engineer stress-tolerant microbes to convert all available sugars to the desired products by routing cellular resources toward product formation and away from cell growth and other unnecessary physiological responses [4].

Several labs have evolved or engineered yeast for anaerobic xylose usage, introducing either xylose isomerase [5–7] or xylose reductase (XR) with xylitol dehydrogenase (XDH) [8–13], and over-expressing xylulokinase for increased flux [14–17]. However, most strains do not use xylose unless further evolved through laboratory selection [7,18–21]. In some cases, the genetic basis for evolved improvements in anaerobic utilization are known from sequencing of evolved lines. We previously engineered a stress-tolerant strain with XR and XDH (strain Y22-3) and evolved it for aerobic xylose respiration, producing strain Y127. To enable anaerobic xylose fermentation, Y127 was propagated anaerobically on xylose, generating strain Y128 [21]. Aerobic xylose respiration in the Y127 strain was enabled by null mutations of the Fe-S cluster protein *ISU1* and the osmotic stress response MAP kinase *HOG1*. Maximal anaerobic fermentation in the evolved Y128 strain was facilitated by these mutations plus additional null mutations of the negative regulator of RAS/PKA signaling, *IRA2*, and *GRE3*, an aldose reductase that siphons xylose to xylitol [22]. A subsequent study independently generated an anaerobic-xylose fermenting strain, confirmed the requirement of the *ISU1* deletion, and further found deletion of the upstream HOG pathway regulator *SSK2* improved xylose fermentation [23]. Thus, mutations in these pathways play a generalizable role in anaerobic xylose fermentation across labs and strains.

While mutations that promote xylose utilization are known, the specific roles for each mutation and how the RAS/PKA and HOG pathways intersect to enable anaerobic xylose utilization remain unclear. RAS signaling promotes growth on preferred nutrients like glucose, in part by activating adenylate cyclase to produce cAMP, which binds to the PKA negative regulatory subunit Bcy1 to enable PKA activity [24]. Ira1/2 are the GTPase activating proteins (GAPs) that inhibit Ras1/2 by converting GTP (RAS-active state) to GDP (RAS-inactive state). On the other hand, Hog1 is best characterized as an osmotic stress response MAP kinase and leads to the upregulation of stress-responsive transcription factors and other enzymes and defense systems [25]. How Hog1 contributes to xylose fermentation is unknown, although the kinase was recently shown to play a role in the response to glucose levels [26–30]. PKA and Hog1 have opposing roles on the stress response: PKA activates transcription factors required for growth-promoting genes and directly suppresses stress-activated transcription factors like Msn2/Msn4, while Hog1 activity induces stress-defense regulators and contributes to the repression of growth-promoting genes [31].

Increased stress sensitivity is a major limitation for industrial use of evolved strains with RAS/PKA and HOG mutations and a barrier to sustainable lignocellulosic bioenergy production. Chemical pretreatment of plant biomass is required to release fermentable sugars into the resulting “hydrolysate.” This treatment produces a variety of toxins and stressors that limit microorganisms’ ability to ferment, particularly impacting fermentation and growth during xylose consumption [2,32,33]. One group of toxins are lignocellulosic hydrolysate inhibitors (or lignotoxins), which are released from breakdown of hemicellulose and cellulose and include furans, phenolics, and aliphatic acids. Lignotoxins disrupt central carbon metabolism pathways by generating reactive oxygen species and depleting the cells of ATP, NADH, and NADPH, in part through increased activity of ATP-dependent efflux pumps and detoxification [34–36], ultimately decreasing available resources for growth and metabolism. Therefore, strains must be tolerant to the toxins present in hydrolysate for efficient fermentation of lignocellulosic material, but the mutations required for efficient anaerobic xylose fermentation produce stress-susceptible strains. Upregulated PKA activity suppresses stress-defense pathways [37–40], while *HOG1* deletion decreases cells’ ability to mount a stress response. This likely has a direct impact on existing strains, limiting their industrial use. One possible solution is increasing stress tolerance in these strains will enable better anaerobic xylose fermentation in industrially relevant hydrolysates. Other groups have found varying levels of success improving toxin tolerance [3] through overexpression of stress response transcription factors [41,42], mitochondrial NADH-cytochrome b5 reductase [42], an oxidative stress protein kinase [43], and furaldehyde reductases [44], as well as through mating, gene shuffling, and evolution [45–48].

We recently discovered an alternate strategy of enabling anaerobic xylose fermentation, one that we predicted may augment stress tolerance. Perturbing sequence of the PKA regulatory subunit Bcy1 through simple protein fusion (with either a 260 amino acid auxin-inducible degron (AiD) tag without degradation capabilities or merely GFP) promotes anaerobic xylose fermentation equal to strain Y128 without the need for *HOG1* or *IRA2* deletion [49]. Since this strain retains functional Hog1 and grows well on glucose, we predicted the strain may have improved stress tolerance, which could enhance growth and xylose fermentation in 9% AFEX-pretreated corn stover hydrolysate (ACSH).

Here, we set out to dissect the contributions of regulators in the RAS/PKA and HOG pathways to anaerobic xylose fermentation, growth, and stress resistance. We demonstrate that while upregulating PKA is important for increased cell growth on glucose, *HOG1* deletion specifically benefits xylose utilization, in part by preventing phosphorylation of glycolytic enzymes by Hog1. Our results show perturbing Bcy1 sequence by protein fusion dramatically improved stress tolerance, as seen by increased growth in toxic 9% glucan-loading ACSH, but xylose fermentation remained blocked, suggesting physiological stress sensitivity is unlikely the cause of halted xylose consumption in concentrated hydrolysate.

## Methods

### Strains and media

Strains used in this study are listed in Table 1. All strains expressed a codon-optimized cassette containing *XYLA* from *Clostridium phytofermentans*, *XYL3* from *Scheffersomyces stipitis*, and *TAL1* from *S*. *cerevisiae* [21]. Generation of strains Y22-3, Y128, Y184, Y184 *ira2Δ*, Y184 *hog1Δ*, Y184 *ira2Δhog1Δ*, Y184 *bcy1Δ*, and 184 Bcy1-AiD was previously described [21,22,49]. Y184 *ira2Δbcy1Δ* was made by replacing *BCY1* with *KanMX* marker in Y184 *ira2Δ*, and verified with diagnostic PCRs. *HOG1* was complemented in Y184 *ira2Δhog1Δ* on a low-copy MoBY 1.0 plasmid [50]. Site-directed mutagenesis was performed to express the *hog1*^*D144A*^ or *hog1*^*A844Δ*^ allele from the MoBY 1.0 plasmid, and correct mutants were verified by sequencing. Strains harboring *HOG1*, *hog1*^*D144A*^, or *hog1*^*A844Δ*^ expressing plasmids or the empty-vector control were grown in media containing G418 to maintain the plasmid.

**Table 1:** Strains used in this study.

Strain ID	Genotype	Reference
Y22-3	NRRL YB-210 MATa spore *HOΔ*::*ScTAL1-CpxylA-SsXYL3-loxP-KanMX-loxP*	[21]
Y128	Y22-3 MATa, *isu1C412T*, *hog1A844del*, *gsh1G839A*, *gre3G136A*, *ira2G8782T*, *sap190A2590G*	[21]
Y184	Y22-3 MATa, *isuΔ*::*loxP-Hyg-loxP*, *gre3Δ*::*MR*	[49]
Y184 *ira2Δ*	Y184, *ira2Δ*::*MR*	[22]
Y184 *bcy1Δ*	Y184, *bcy1Δ*::*KanMX*	[49]
Y184 *hog1Δ*	Y184, *hog1Δ*::*KanMX*	[22]
Y184 Bcy1-AiD	Y184, BCY1-3' AiD tag (3x Mini-Auxin Induced Degron Sequence-5x FLAG-BCY1-3' UTR-KanMX) (G418R)	[49]
Y184 *ira2Δhog1Δ*	Y184, *ira2Δ*::*MR*, *hog1Δ*::*KanMX*	[22]
Y184 *ira2Δbcy1Δ*	Y184, *ira2Δ*::*MR*, *bcy1Δ*::*KanMX*	This study
Y184 *ira2Δhog1Δ/HOG1*	Y184 *ira2Δhog1Δ*, *HOG1 (*CEN4 plasmid: *KanMX*, *URA3*)	This study, [50]
Y184 *ira2Δhog1Δ/hog1D144A*	Y184 *ira2Δhog1Δ*, *hog1D144A (*CEN4 plasmid: *KanMX*, *URA3*)	This study, [50]
Y184 *ira2Δhog1Δ/A884Δ*	Y184 *ira2Δhog1Δ*, *hog1A844del (*CEN4 plasmid: *KanMX*, *URA3*)	This study, [50]
Y184 *ira2Δhog1Δ/*empty	Y184 *ira2Δhog1Δ*, CEN4 plasmid: *KanMX*, *URA3*	This study, [50]
Y184 *ira2Δ/*empty	Y184 *ira2Δ*, CEN4 plasmid: *KanMX*, *URA3*	This study, [50]

YPDX medium was prepared as previously described [51] (1% yeast extract, 2% peptone, except that sugars were added at either 6% glucose and 3% xylose or 9% glucose and 4.5% xylose). Identified lignotoxins [52,53] (S1 Table) were added to YPDX 6%/3% and the medium was sterilized by vacuum filtration. ACSH was prepared from *Zea mays* (Pioneer hybrid 36H56) grown in Field 570-C Arlington Research Station, University of Wisconsin and harvested in 2012, as previously described [54]. Pretreated corn stover was hydrolyzed to either 6% or 9% glucan loading at 50°C for 5 days, and biomass was added 4 hours after hydrolysis began in 4 batches. The hydrolysate was centrifuged (2500xg for 30 minutes) and sterile filtered (0.22 μm pore size; Millipore Stericup). Final sugar concentrations were 53 g/L glucose and 21.7 g/L xylose for 6% glucan-loading ACSH, and 80 g/L glucose and 36 g/L xylose for 9% glucan-loading ACSH. Osmolality of media was measured with a Wescor VAPRO® Vapor Pressure Osmometer 5600.

### TECAN screening

Strains were grown to saturation in YPD 2% batch aerobically overnight, then diluted to an OD_600_ of 2 in YPD 2%. 5 μL of culture was added to 95 μL of 9% ACSH, YPDX 9%/4.5%, YPDX 6%/3%, or YPDX 6%/3% +LT in a Costar clear 96-well plate. OD at 600 nm was measured anaerobically for 48 hours in a TECAN Infinite 200 at 30°C, with measurements taken every 20 minutes (total of 144 measurement cycles) and multiple reads per well. Final OD was calculated based on the average OD reading per well per time point and using three biological replicates. Significant differences compared to Y184 were determined by performing a paired T-test at *p* < 0.05, pairing sample by replicate date.

### Batch culture fermentations

Overnight aerobic cultures grown in YPD 2% glucose were transferred to an anaerobic chamber, washed once with anaerobic medium, and inoculated into the tested media at an OD_600_ of 0.1. Fermentations occurred for 96 hours, with 1 mL aliquots removed throughout the time course for OD_600_ measurements and HPLC-RID analysis to measure glucose, xylose, and ethanol concentrations. Dry-cell weight biomass was measured by vacuum filtering cultures onto pre-weighed filters (0.45 μm pore size), microwaving on 10% power for 10 minutes, then drying in a desiccant for 24 hours before measuring. Biomass, OD, and concentrations of glucose, xylose, and ethanol were averaged from three biological replicates for each time point. ANOVA was used to determine significant differences in biomass, comparing 24h and 48h within each individual strain, at *p* < 0.05. Rates of sugar consumption were calculated by normalizing the change in sugar concentration to the fitted rate of biomass change during exponential (glucose) or stationary (xylose) phase, and a paired T-test was used to determine significant differences compared to Y184 at *p* < 0.05. Ethanol titer at 48h was averaged from three biological replicates.

### Phosphoproteomic analysis

Quantitative proteomic and phosphoproteomic samples of Y184 *ira2Δhog1Δ* and Y184 *hog1Δ* were prepared as previously described using isobaric tandem mass tags (TMT) for phosphoproteomic analysis [49]. Paired samples were collected from two independent replicates grown anaerobically in YPX 3% for three generations and harvested at OD_600_ ~0.5. Proteomic samples were analyzed by nanoflow liquid chromatography tandem mass spectrometry. COMPASS [55] was used to search against target-decoy yeast database [56]. Raw data were transformed to log_2_ values, then the fold change between Y184 *ira2Δhog1Δ* and Y184 *ira2Δ* peptides was calculated. We focused on differentially abundant phosphopeptides, defined as those with a log_2_ difference of at least 1.5X in the same direction in both replicate comparisons. We used this threshold since TMT tagging is known to compress abundance differences [57].

## Results

**Fig 1:**
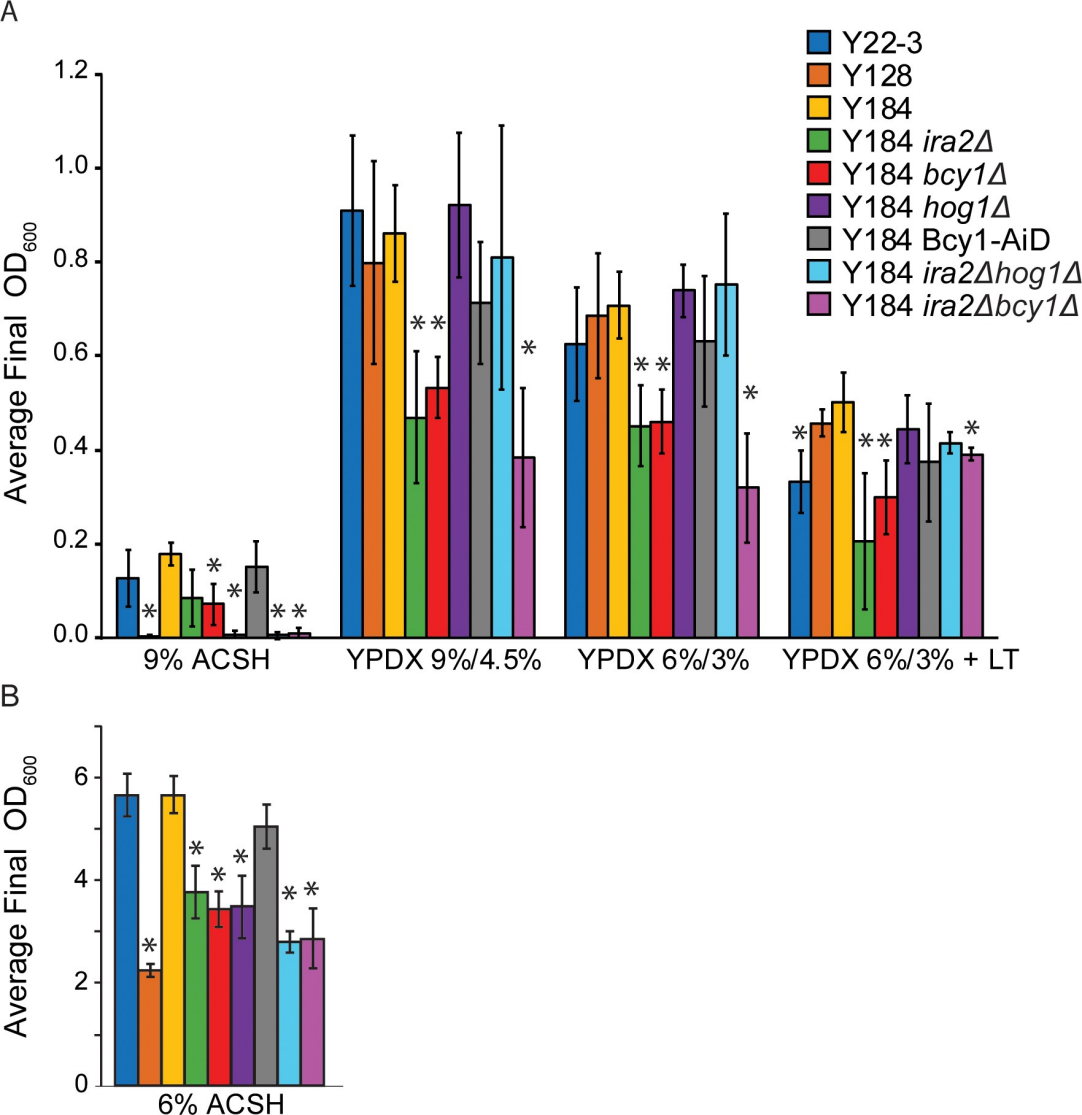
Stress susceptibility of strains engineered for xylose consumption. **A)** Strains were grown anaerobically in 9% ACSH, YPDX 9%/4.5%, YPDX 6%/3%, and YPDX 6%/3% + lignotoxins (LT) in 96-well plates, and final cell density at 48h was measured with a TECAN instrument. **B)** Same as A) except cells were grown in batch culture and OD was measured at 32 hours. Average and standard deviations of final ODs were calculated from three biological replicates. Asterisks denote significant differences compared to Y184 grown in each respective condition (paired T-Test, *p* < 0.05).

Our goal was to dissect the contributions of different regulators in growth, xylose fermentation, and stress tolerance. We therefore measured fermentation and growth rates of a panel of strains grown in several concentrations of ACSH and laboratory media. To clarify the contribution of each regulator, we started with strain Y22-3, which overexpresses a codon-optimized cassette containing *XYLA* from *Clostridium phytofermentans*, *XYL3* from *Scheffersomyces stipitis*, and *TAL1* from *S*. *cerevisiae* but cannot utilize xylose, and strain Y184, which additionally lacks *ISU1* and *GRE3* and can thus respire xylose aerobically but not anaerobically [49]. Both Y22-3 and Y184 can grow in the toxic 9% ACSH, whereas strain Y128, which can ferment xylose anaerobically, cannot (Fig 1). To test the impact of mutations that enable anaerobic xylose utilization, we generated Y184 derivatives lacking *IRA2*, *BCY1*, or *HOG1* individually as well as *IRA2 HOG1* or *IRA2 BCY1* in combination, to define how each mutation impacts stress tolerance, growth, and metabolism. We also included Y184 Bcy1-AiD, in which an auxin-inducible degradation sequence is fused to the C-terminal of Bcy1 (in the presence of functional *IRA2* and *HOG1*); it is important to note this sequence alone does not enable degradation of Bcy1 but imparts rapid xylose fermentation in lab medium without the need for *HOG1* deletion [49].

### Improving stress tolerance does not restore xylose fermentation in toxic hydrolysate

We first measured tolerance of each strain to ACSH hydrolysates and rich laboratory medium containing mixed glucose/xylose with and without lignotoxins (LT), anaerobically in a 96-well plate reader. 9% ACSH is clearly inhibitory to growth, since all strains grew to much lower cell densities than in rich medium with lignotoxins added (Fig 1). Parental strains Y22-3 and Y184 showed the best growth in 9% ACSH, as indicated by final cell density, even though they cannot use the xylose after glucose is consumed. Y184 *ira2Δ* and Y184 *bcy1Δ* showed reduced growth compared to Y184, but still grew; Y128, Y184 *hog1Δ*, Y184 *ira2Δhog1Δ*, and Y184 *ira2Δbcy1Δ* were not able to grow. Thus, all strains lacking functional Hog1 showed increased sensitivity to 9% ACSH, as did the Y184 *ira2Δbcy1Δ* strain. Since Hog1 functions in the osmotic stress response, we reasoned the high sugar content of 9% ACSH may be responsible for the sensitivity of *hog1Δ* strains. However, the mutants were not sensitive to rich medium with sugar concentrations matching 9% ACSH (9% glucose, 4.5% xylose, Fig 1A). 9% ACSH is more concentrated than YPDX with 9% glucose / 4.5% xylose (1922 mmol / kg for 9% ACSH versus 1156 mmol / kg for YPDX). Yet, the mutants also showed increased sensitivity to 6% ACSH (Fig 1B), whose osmolality is on par with sugar-supplemented rich medium (1225 mmol / kg). Together, this suggests that it is not only the osmolality of 9% ACSH that is inhibitory to these strains. Strikingly, Y184 cells with the Bcy1-AiD tag, which enables anaerobic xylose fermentation, displayed maximal growth in 9% ACSH, comparable to Y184. Therefore, as we predicted, Y184 Bcy1-AiD lacks the extreme stress sensitivity seen in *hog1Δ* strains. While adding lignotoxins to YPDX medium decreased growth, it was not as inhibitory to any strain as 9% ACSH, suggesting either the lignotoxin cocktail [53] added was lower than toxin levels in real hydrolysate, that additional toxins not in our cocktail remain to be identified, or that it is the combination of osmolarity and toxins that contributes to toxicity.

**Fig 2:**
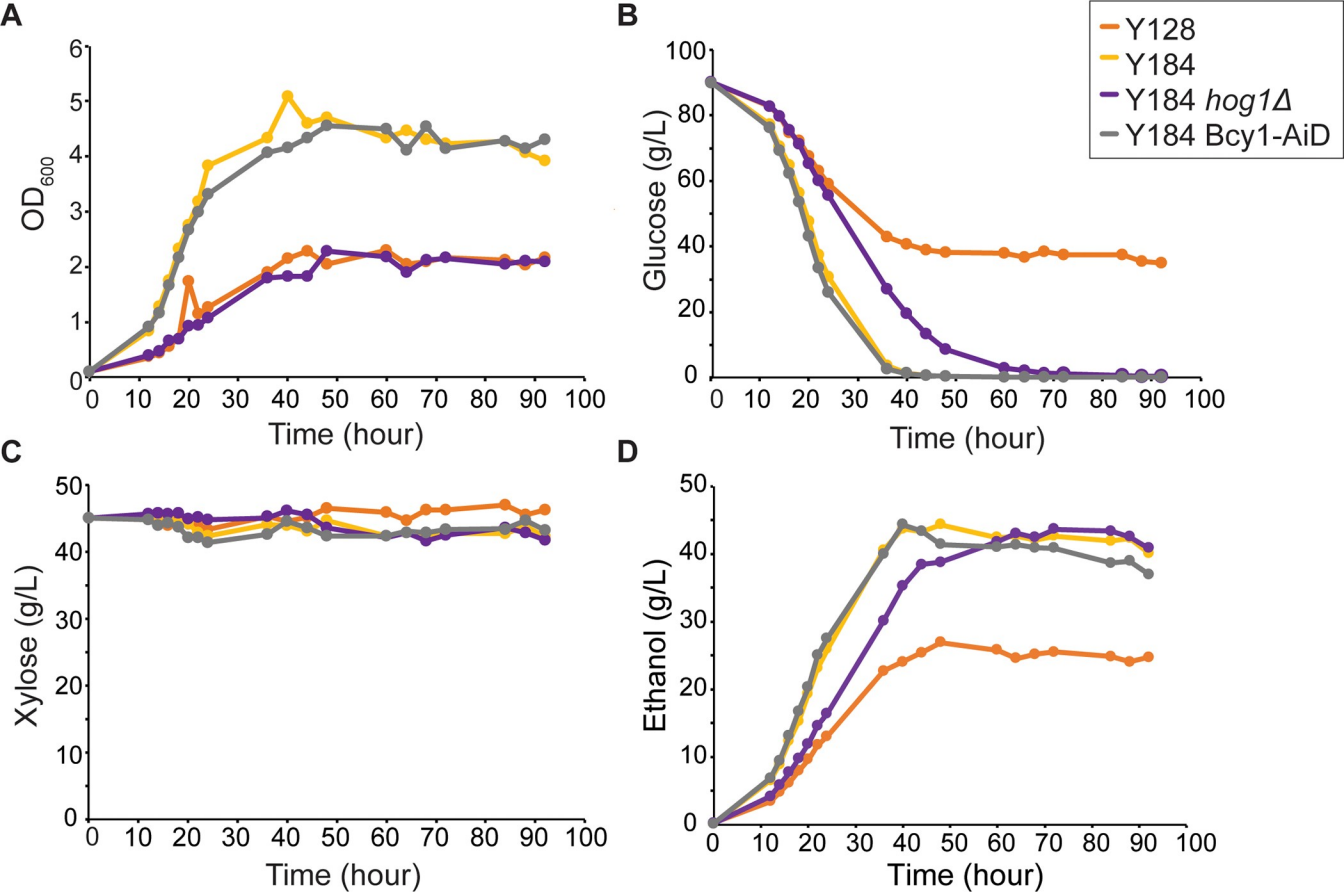
9% ACSH is inhibitory to xylose fermentation. Batch cultures of Y128, Y184, Y184 *hog1Δ*, and Y184 Bcy1-AiD were grown anaerobically for 92 hours in 9% ACSH. **A.** Average OD_600_ measurements, **B.** glucose concentration, **C.** xylose concentration, and **D.** ethanol concentration over time, from three biological replicates.

We next studied glucose and xylose consumption in 9% ACSH to characterize fermentation rates. We expected the strains whose growth was sensitive to the stresses of ACSH would ferment worse compared to tolerant strains. Surprisingly, we found all strains, even those containing functional Hog1, were incapable of fermenting xylose in hydrolysate generated at high-glucan loading (Fig 2C, S1C Fig). Y128 and Y184 *hog1Δ* did not grow to densities as high as Y184, although Y184 *hog1Δ* was able to ferment the glucose and produce ethanol (Fig 2D). Y184 Bcy1-AiD showed division as robust as Y184 (Fig 2A), supporting observations seen with the 96-well plate screen. Consistent with our hypothesis, Y184 *hog1Δ* and especially Y128 cultures showed reduced glucose consumption over the time course, whereas stress-tolerant Y184 and Y184 Bcy1-AiD depleted the glucose by 40 hours (Fig 2B). Since we previously showed the Y184 Bcy1-AiD strain can ferment xylose anaerobically at higher rates than Y128 in laboratory medium [49] and has dramatically improved growth in 9% ACSH, we predicted Y184 Bcy1-AiD would display enhanced xylose consumption compared to Y128 and Y184 *hog1Δ* in 9% ACSH. Unexpectedly, this was not observed, as none of the strains used xylose in 9% ACSH (Fig 2C) even though two fermented the glucose. Several strains marginally used xylose in 6% ACSH (S2C Fig), but clearly ferment xylose in YPDX (see below), this suggests stresses in ACSH medium prevent xylose fermentation. Since the growth and glucose consumption of Y184 Bcy1-AiD is clearly recovered, these results suggest cellular stress is unlikely to be the cause of arrested xylose fermentation (see Discussion).

### Differential contributions of regulators to glucose versus xylose fermentation rates

**Fig 3:**
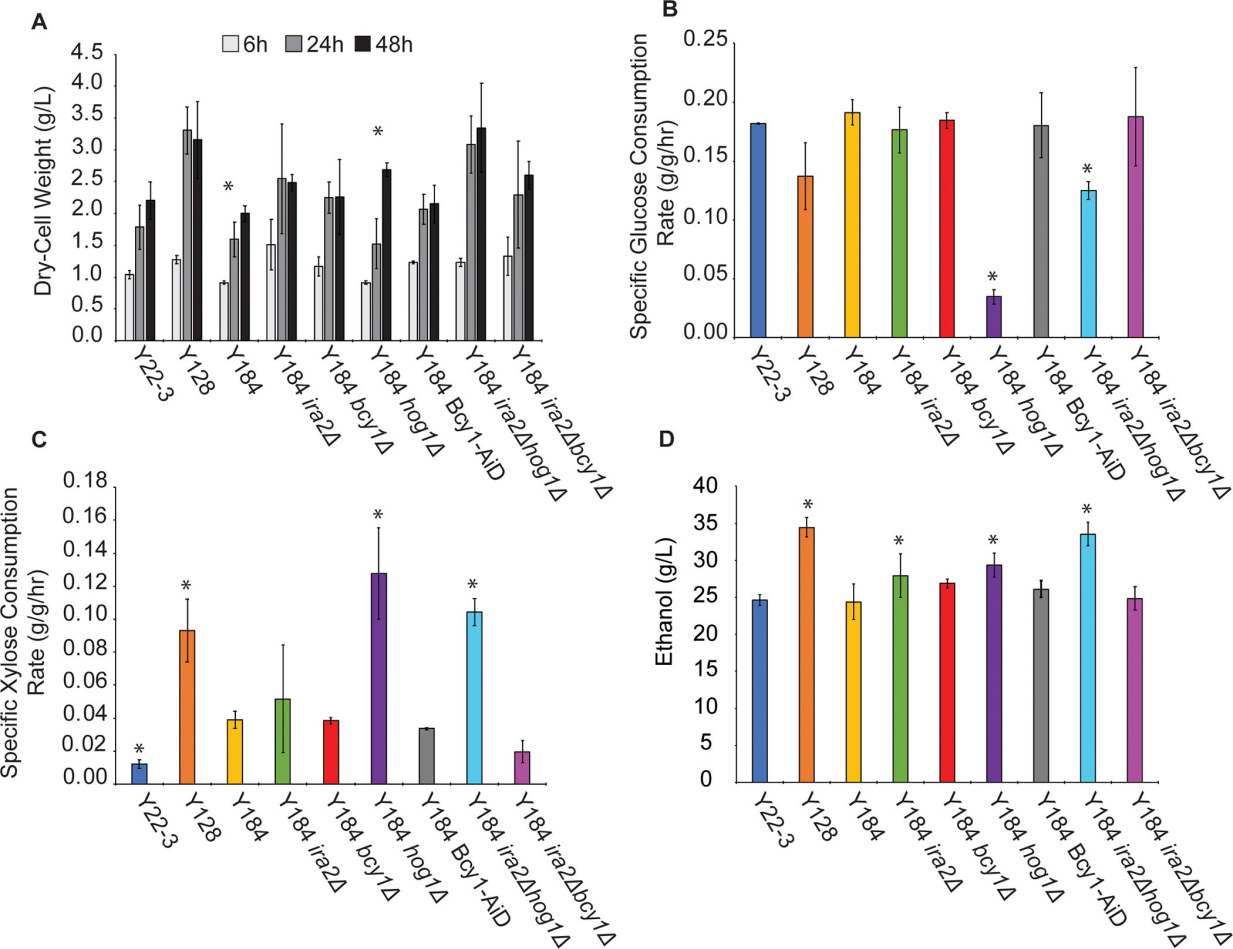
PKA activity increases growth, while *hog1Δ* improves xylose consumption. Batch cultures were grown in YPDX 6%/3% for 96 hours. Measurements are averages from three biological replicates. **A.** The average dry-cell weight biomass (g per L culture) measured at 6, 24, and 48 h post inoculation. Asterisk indicates a significant difference (p< 0.055) between the 24h and 48h time point within each strain. For all strains except Y184 and Y184 *hog1Δ*, the biomass accumulated was not significantly different at 48h compared to 24h, indicating faster saturation of those cultures. **B.** Specific glucose consumption rates calculated from the exponential phase of growth. **C.** Specific xylose consumption rate calculated from the stationary phase of growth. **D.** Ethanol titer at 48 hours post inoculation. For **B,C,D**, asterisks denote significant differences compared to Y184 (paired T-Test, *p* < 0.05).

We next wanted to dissect the contribution of Ira2, Bcy1, and Hog1 to sugar fermentation. We worked with laboratory media supplemented with 60 g/L glucose and 30 g/L xylose to characterize fermentation when the strains are most productive, since they did not ferment xylose efficiently in ACSH. Interestingly, strains lacking Ira2 or Bcy1 or harboring the Bcy1-AiD fusion protein showed faster growth compared to the parental strains: all strains with these mutations reached maximum cell density by 24h, whereas Y22-3, Y184, and Y184 *hog1Δ* took 48h to reach maximal titers (Fig 3A, S3A Fig). Although it was not statistically significant in this growth assay, we noticed in the 96-well plate assay (Fig 1) strains lacking *IRA2* or *BCY1* consistently grew to lower cell densities than the parental strains. Deletion of *HOG1* from Y184 reduced growth rate and glucose consumption rate compared to the other strains, whereas combined deletions of *HOG1* and *IRA2* produced a strain with fast growth rates and the ability to grow to a higher cell density than Y22-3 and Y184 (Fig 3A and 3B and S3A and S3B Fig). Thus, the beneficial effect of *IRA2* deletion overrides the deleterious effect of *HOG1* deletion for glucose-based growth.

While mutations in PKA regulators increase growth on and consumption of glucose, the opposite was observed for xylose consumption. Strains lacking *HOG1* had the highest rates of xylose consumption compared to the parental strains, whereas strains with PKA mutations alone did not have a significantly different xylose consumption rate from the Y184 parental strain (Fig 3C). Thus, mutating *HOG1* is specifically important for efficient xylose fermentation. Moreover, Y128, Y184 *hog1Δ*, and Y184 *ira2Δhog1Δ* begin to ferment the xylose before glucose is completely depleted from the culture (S3B and S3C Fig). Correspondingly, Y128, Y184 *hog1Δ*, and Y184 *ira2Δhog1Δ* had the highest ethanol titer at 48 hours (Fig 3D, S3D Fig). Y184 Bcy1-AiD was previously shown to use xylose at a comparable rate to Y128 in YPX medium when cultured at a high cell density [49]. Interestingly, we discovered that the strain does not use xylose efficiently when the culture is started at low cell density (Fig 3C), even though we recapitulate robust xylose utilization at high cell density in YPDX (S4A Fig). The reason for this unique phenotype is not known; importantly, it did not explain the lack of xylose consumption in ACSH, since inoculating cultures at high cell titers did not improve xylose fermentation in the strain (S4B Fig).

### Hog1 kinase activity decreases xylose fermentation

**Fig 4:**
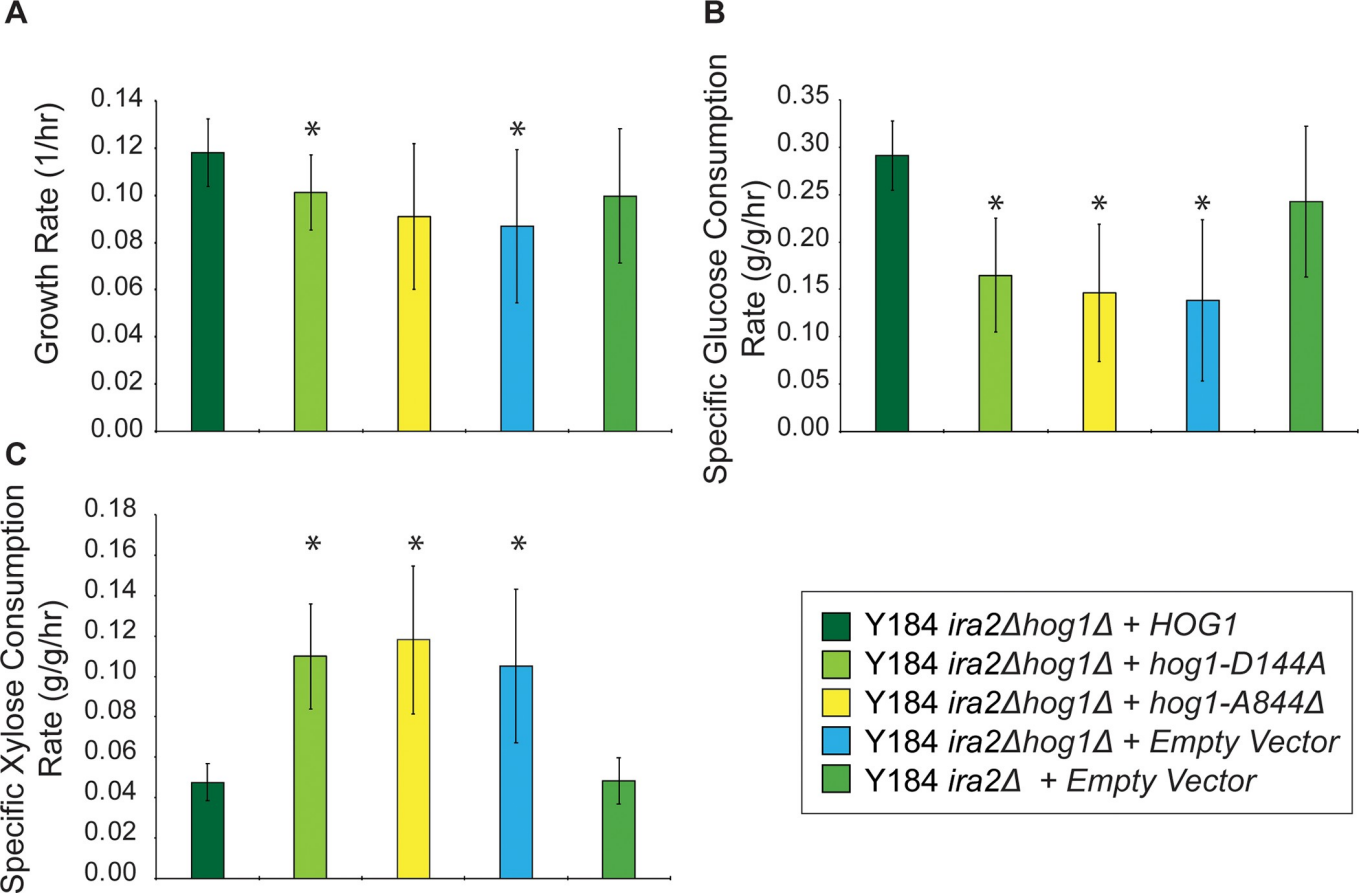
Hog1 activity blocks xylose consumption. Wildtype, catalytically inactive (*hog1*^*D144A*^), and truncated (*hog1*^*A844𝞓*^) *HOG1* alleles were expressed in Y184 *ira2Δhog1Δ* cells and grown in batch culture for 96 hours in YPDX 6%/3%. Rate measurements were based on average and standard deviation from three biological replicates. **A.** Average growth rate calculated from the exponential phase of growth. **B.** Specific glucose consumption rate calculated from the exponential phase of growth. **C.** Specific xylose consumption rate calculated from the stationary phase of growth. Asterisks denote significant differences from Y184 *ira2Δhog1Δ*/*HOG1* (paired T-Test, *p* < 0.05).

*HOG1* deletion allows for efficient xylose fermentation in YPDX. To test if it is the kinase activity of Hog1, as opposed to other effects of the Hog1 protein, we tested if the catalytically inactive *hog1-D144A* allele or truncated *HOG1* recapitulating Y128’s mutation (*hog1 A844Δ*) could block xylose fermentation when introduced into Y184 *ira2Δhog1Δ*, equal to reintroducing the functional *HOG1* allele. We saw the catalytic activity of Hog1 was required to suppress anaerobic xylose fermentation: reintroducing wildtype *HOG1* increased glucose consumption and inhibited xylose fermentation, whereas expression of catalytically inactive or truncated Hog1 did not significantly affect the glucose or xylose consumption rate, since the strains matched the performance of Y184 *ira2Δhog1Δ* (Fig 4). This reveals Hog1 kinase activity normally inhibits xylose fermentation, suggesting Hog1 actively phosphorylates one or more targets to prevent xylose utilization.

We performed phosphoproteomic analysis to implicate potential Hog1 targets impacting xylose consumption. Y184 *ira2Δ* and Y184 *ira2Δhog1Δ* were grown anaerobically in batch culture in YPX medium, and phospho-proteomes were measured by quantitative mass-spectrometry (see Methods). We identified only 11 phosphopeptides whose abundance was reproducibly lower in Y184 *ira2Δhog1Δ* (Table 2, see methods). Of these 11, four are linked to metabolism: E1 alpha subunit of pyruvate dehydrogenase (Pda1), pyruvate kinase (Cdc19), glyceraldehyde-3-phosphate dehydrogenase (Tdh1), and trehalose-6-phosphatase complex (Tsl1). Pda1, Cdc19, and Tdh1 have roles in glycolysis, whereas Tsl1 generates the overflow metabolite trehalose. In contrast, eight phosphopeptides showed reproducibly increased abundance in Y184 *ira2Δhog1Δ*. Of these eight, four are from proteins with unknown functions, one is a heat shock protein (Hsp26), two are components of the 40S ribosome (Rps6A, Rps6B), and one is an epsin-like protein involved in endocytosis (Table 2). Several of these enzymes, including Cdc19 and Tsl1, directly interact with Hog1, and others harbor the known Hog1 consensus sites around the affected residue, suggesting Hog1 directly phosphorylates these proteins (see Discussion).

**Table 2:** Significant phosphorylation changes in Y184 *ira2Δhog1Δ*.

Protein	Function	Phospho-site	Average Log2(fold change) in Y184 *ira2Δhog1Δ versus Y184 ira2Δ*
PDA1	Pyruvate dehydrogenase	Y344	-1.66
EIS1	Eisosome protein	T767	-1.44
PDA1	Pyruvate dehydrogenase	S336	-1.16
EIS1	Eisosome protein	S763	-1.14
TSL1	Trehalose 6-phosphate synthase/phosphatase	S266	-1.10
HBT1	Shmoo tip protein	S41	-1.08
MRP51	Mitochondrial small ribosomal protein	S327	-1.03
CDC19	Pyruvate kinase	S22	-0.93
TDH1	Glyceraldehyde-3-phosphate dehydrogenase	T136	-0.84
RPS21B	40S ribosomal subunit	S65	-0.74
HBT1	Shmoo tip protein	S363	-0.74
ENT1	Epsin-like protein	S317	0.68
INA1	Unknown function	S652;S657	0.76
YNL115C	Unknown function	S244	0.79
INA1	Unknown function	S654;S657	0.86
YNL115C	Unknown function	S42	1.00
HSP26	Small heat shock protein	S208	1.02
RPS6B	40S ribosomal subunit	S232	1.14
RPS6A	40S ribosomal subunit	S232	1.14

## Discussion

Our results provide new insights into how mutation of different signaling proteins can impart distinct physiological responses that, when integrated, improve anaerobic conversion of sugars to products, in this case ethanol. We show mutations in RAS/PKA signaling that up-regulate PKA activity enhance growth and perhaps glucose consumption rates, but possibly at a cost of final cell density, whereas deletion of HOG1 is essential for xylose fermentation but at the expense of hydrolysate tolerance. Together, these results suggest several important results relevant to lignocellulosic fermentation.

PKA and Hog1 contribute separable features to xylose fermentation. Increased PKA activity is known to increase cell growth rate and glucose consumption [58]. PKA upregulates expression of ribosome biogenesis genes, which supports rapid growth [59,60]. Moreover, it promotes glycolytic flux by inducing transcription of genes and phosphorylating enzymes involved in glycolysis [61–63]. We recently showed PKA also has roles in the hypoxic response when cells are grown anaerobically on xylose [49]. This response is mediated by the Azf1 and Mga2 transcription factors and may further promote glycolytic flux. Therefore, it is consistent that we find strains with deletions of RAS/PKA regulators display faster growth (Fig 3A, S3A Fig). Despite the increased growth and glucose consumption rates when PKA regulators are deleted, this effect is not as dramatic with xylose. While strains with mutations in *IRA2* or *BCY1* do consume more xylose than Y184 (S3C Fig), the rates of consumption are not significantly faster than Y184 (Fig 3C). The reduced final-cell titers seen in strains with up-regulated RAS/PKA may result from an inability to accumulate storage carbohydrates. During the diauxic shift, glycogen storage occurs when glucose has been depleted to fifty percent of its starting concentration [64]. However, increased PKA activity prevents accumulation of storage carbohydrates, such as glycogen and trehalose, by inhibiting expression of biosynthesis enzymes and activating catabolic enzymes [65–69]. In our strains lacking RAS/PKA inhibitors, it is possible upon glucose depletion, the cells lack stored carbohydrates to metabolize, limiting cell titers compared to strains with functional *IRA2* and *BCY1* (Fig 1, S3A Fig).

In contrast to PKA upregulation, *HOG1* deletion specifically affects xylose utilization. Our results show that under standard conditions, even in the absence of added stress, Hog1 kinase activity inhibits xylose utilization, perhaps by directly phosphorylating glycolytic enzymes (Fig 4C, Table 2). Although typically thought of as a stress regulator, Hog1 has recently been implicated in the response to glucose [28,29,70] and was shown to phosphorylate the glycolytic enzyme Pfk26 [71] and physically interact with Cdc19, Pfk1, and Pfk2 [72]. Hog1 also plays an important role in glycerol production, which may influence glycolytic flux to steer production from pyruvate towards glycerol [73–78]]. The enzymes we detected with significant phosphorylation differences upon *HOG1* deletion function lower in glycolysis, after the entry point of xylose via the pentose phosphate pathway (Table 2), but we cannot exclude that other enzymes or regulators are also affected. Modeling of glycolytic flux during osmoadaption suggests Hog1 activity stabilizes pyruvate production to prevent starvation [76], which may account for the decreased phosphorylation of Cdc19’s activation site in *hog1Δ* mutants (Table 2).

An interesting question is the relationship between PKA and Hog1 activity. We previously found Y184 *bcy1Δ* cells display reduced phosphorylation of the Hog1 protein on its activating site when grown anaerobically in YPX medium [49], suggesting a PKA-dependent mechanism of inhibiting Hog1 activity to allow xylose fermentation. Hog1 was shown to be activated during glucose depletion [28,70]. It is possible as cells begin to deplete glucose from YPDX, Hog1 becomes activated in Y184 but that this is suppressed when PKA is up-regulated via *BCY1* or *IRA2* deletion. The largest effects on xylose metabolism occur when PKA upregulation and *HOG1* deletion are combined. Unfortunately, a byproduct of this is likely causing extreme stress sensitivity. Strains lacking *HOG1* (Y128, Y184 *hog1Δ*, and Y184 *ira2Δhog1Δ*) are especially sensitive to hydrolysate (Figs 1 and 2A, S1A Fig). Moreover, the combination of upregulated PKA and *HOG1* deletion may exacerbate stress sensitivity, since PKA suppresses the stress response [24], while Hog1 activates it [25]. These strains are able to ferment xylose in favorable conditions (Fig 3C, S3C Fig), but are unable to ferment the sugar in toxic 9% ACSH (Fig 2C, S1C Fig).

We hypothesized improving stress tolerance would improve xylose fermentation in stressful conditions. We predicted Y184 Bcy1-AiD, which harbors functional *HOG1* but can ferment xylose anaerobically in rich medium when started at high titers [49], would display both higher stress tolerance and anaerobic xylose fermentation in hydrolysate. Interestingly, while Y184 Bcy1-AiD clearly grew well in 9% ACSH, it did not utilize the xylose even when started at high cell titer (Figs 1, 2A and 2C, S4B Fig). This suggests stress sensitivity is not the sole factor limiting xylose fermentation. There are many types of lignotoxins present in hydrolysates [52], and while their effects are somewhat understood, many of their specific targets remain to be elucidated [33]. One possibility is that toxins are directly inhibiting enzymes required for anaerobic xylose fermentation, as shown in *Escherichia coli* where feruloyl and coumaroyl amides were discovered to be allosteric inhibitors of *de novo* nucleotide biosynthetic enzymes [79]. Jayakody et al. (2018) found that glycoaldehyde and methyglyoxal present in hydrolysate are key inhibitors of xylose fermentation [80]. Furthermore, acetic acid is known to decrease enolase activity, ultimately slowing down glycolysis [81]. This has led to other groups engineering yeast to reduce and ferment acetate or increase acetate tolerance [43,82,83]. More studies identifying the targets of lignotoxins will help to clarify the bottleneck in xylose metabolism when microorganisms are grown in hydrolysate media.

Our results also revealed unexpected information on the different routes of PKA regulation with regard to the stress response. Removing either *IRA2* or *BCY1* had mild impacts on ACSH tolerance, but deletion of both genes greatly impacted tolerance of 9% ACSH (Figs 1 and 2A, S1A Fig). This suggests there are multiple lines of partially redundant regulation of stress tolerance by the RAS/PKA pathways. Upregulation RAS by *IRA2* deletion is predicted to increase cAMP and thus PKA activity [84], whereas deletion of *BCY1* removes the cAMP-responsive inhibitor of PKA. One possibility is PKA is only partially upregulated by single-gene deletion, but double-deletion of *IRA2* and *BCY1* produces much stronger activation and thus complete suppression of the stress response. There are other methods of RAS/PKA pathway regulation which support the possibility that single deletion of *IRA2* or *BCY1* causes only a partial upregulation. The RAS/PKA pathway undergoes feedback inhibition to control cAMP concentrations through predicted PKA-directed phosphorylation of Cdc25 and Pde1 [85]. Furthermore, the interaction between the catalytic and regulatory subunits of PKA is regulated by other factors, including kelch-repeat proteins Krh1/2 [86]. Another possibility is PKA may be directed to different substrates in different situations. Beyond allosteric cAMP regulation influenced by RAS, Bcy1 is also regulated by phosphorylation, which can influence PKA substrate specificity and localization [87–91]. Recent studies in mammalian cells show the negative regulator of PKA does not dissociate from the active kinase at physiological cAMP levels [92], and the substrate determines the dissociation rate of catalytic and regulatory subunits [93]. Thus, activation of PKA via Bcy1-cAMP binding may provide different effects than if Bcy1 is missing from the cell. Finally, it is also possible that PKA-independent effects of *IRA2* deletion separately regulate the stress response. Future studies to dissect these and other effects will contribute to our understanding of how to engineer cells for anaerobic xylose fermentation in lignocellulosic hydrolysates.

## Supporting information

S1 Fig 9% Growth and metabolism profiles in 9% ACSH. Batch cultures were grown in 9% ACSH anaerobically for 92 hours. Data represent average and standard deviation from three biological replicates. **A.** OD_600_ measurements over time. Glucose (**B.**) and xylose (**C.**) concentration in the media over time.
(TIF)

S2 Fig Growth and metabolism profiles in 6% ACSH. As described in S1 Fig except for anaerobic 6% ACSH growth, measuring dry-cell weight (**A.**), and glucose (**B.**), xylose (**C.**), and ethanol (**D.**) media concentration over time.
(TIF)

S3 Fig Growth and metabolism profiles in YPDX 6%/3%. As described in S1 Fig except for anaerobic YPDX 6%/3% growth, measuring dry-cell weight (**A.**), and glucose (**B.**), xylose (**C.**), and ethanol (**D.**) media concentration over time.
(TIF)

S4 Fig High starting cell titers increases xylose consumption in nutrient-rich medium, but not ACSH. Batch cultures were grown anaerobically for 96 hours in YPDX 6%/3% (**A.**) or 6% ACSH (**B.**). Cultures were started at an OD_600_ of 3. Data represent average and standard deviation of three biological replicates. Comparing Panel A to Fig 3C shows that the Y184 Bcy1-AiD strain ferments xylose when the culture is inoculated at a higher starting OD but not when inoculated at a lower cell density.
(TIF)

S1 Table Concentrations of lignotoxins present in 9% ACSH and YPDX 6%/3% + LT. This table lists the concentrations of lignotoxins identified in 9% ACSH and the corresponding concentrations added to generate YPDX 6%/3% +LT.
(XLSX)

## References

[1] van Maris AJA, Abbott DA, Bellissimi E, van den Brink J, Kuyper M, Luttik MAH, et al Alcoholic fermentation of carbon sources in biomass hydrolysates by *Saccharomyces cerevisiae*: current status. Antonie Van Leeuwenhoek. 2006 11 11;90(4):391–418. 10.1007/s10482-006-9085-7 17033882

[2] Klinke HB, Thomsen AB, Ahring BK. Inhibition of ethanol-producing yeast and bacteria by degradation products produced during pre-treatment of biomass. Appl Microbiol Biotechnol. 2004 11 6;66(1):10–26. 10.1007/s00253-004-1642-2 15300416

[3] Caspeta L, Castillo T, Nielsen J. Modifying yeast tolerance to inhibitory conditions of ethanol production processes. Front Bioeng Biotechnol. 2015 11 11;3:184 10.3389/fbioe.2015.00184 26618154 PMC4641163

[4] Deparis Q, Claes A, Foulquié-Moreno MR, Thevelein JM. Engineering tolerance to industrially relevant stress factors in yeast cell factories. FEMS Yeast Res. 2017 6 1;17(4). 10.1093/femsyr/fox036 PMC5812522 28586408

[5] Kuyper M, Harhangi H, Stave A, Winkler A, Jetten M, Delaat W, et al High-level functional expression of a fungal xylose isomerase: the key to efficient ethanolic fermentation of xylose by? FEMS Yeast Res. 2003 10 1;4(1):69–78. 10.1016/S1567-1356(03)00141-7 14554198

[6] Kuyper M, Hartog M, Toirkens M, Almering M, Winkler A, Vandijken J, et al Metabolic engineering of a xylose-isomerase-expressing strain for rapid anaerobic xylose fermentation. FEMS Yeast Res. 2005 2 1;5(4–5):399–409. 10.1016/j.femsyr.2004.09.010 15691745

[7] Demeke MM, Dietz H, Li Y, Foulquié-Moreno MR, Mutturi S, Deprez S, et al Development of a D-xylose fermenting and inhibitor tolerant industrial *Saccharomyces cerevisiae* strain with high performance in lignocellulose hydrolysates using metabolic and evolutionary engineering. Biotechnol Biofuels. 2013 6 21;6(1):89 10.1186/1754-6834-6-89 23800147 PMC3698012

[8] Ho NW, Chen Z, Brainard AP. Genetically engineered *Saccharomyces* yeast capable of effective cofermentation of glucose and xylose. Appl Environ Microbiol. 1998 5 1;64(5):1852–9. 9572962 10.1128/aem.64.5.1852-1859.1998 PMC106241

[9] Jin Y-S, Seo H. Conversion of xylose to ethanol by recombinant *Saccharomyces cerevisiae* containing genes for xylose reductase and xylitol dehydrogenase from *Pichia stipitis*. J Microbiol Biotechnol. 2000 8 1;10(4):564–7.

[10] Kötter P, Amore R, Hollenberg CP, Ciriacy M. Isolation and characterization of the *Pichia stipitis* xylitol dehydrogenase gene, *XYL2*, and construction of a xylose-utilizing *Saccharomyces cerevisiae* transformant. Curr Genet. 1990;18(6):493–500. 2127555 10.1007/BF00327019

[11] Walfridsson M, Hallborn J, Penttilä M, Keränen S, Hahn-Hägerdal B. Xylose-metabolizing *Saccharomyces cerevisiae* strains overexpressing the *TKL1* and *TAL1* genes encoding the pentose phosphate pathway enzymes transketolase and transaldolase. Appl Environ Microbiol. 1995 12 1;61(12):4184–90. 8534086 10.1128/aem.61.12.4184-4190.1995 PMC167730

[12] Jin Y-S, Jeffries TW. Changing flux of xylose metabolites by altering expression of xylose reductase and xylitol dehydrogenase in recombinant *Saccharomyces cerevisiae* In: Biotechnology for Fuels and Chemicals. Totowa, NJ: Humana Press; 2003 p. 277–85. 10.1385/abab:106:1-3:277 12721451

[13] Kim SR, Ha S-J, Kong II, Jin Y-S. High expression of *XYL2* coding for xylitol dehydrogenase is necessary for efficient xylose fermentation by engineered *Saccharomyces cerevisiae*. Metab Eng. 2012 7 1;14(4):336–43. 10.1016/j.ymben.2012.04.001 22521925

[14] Johansson B, Christensson C, Hobley T, Hahn-Hägerdal B. Xylulokinase overexpression in two strains of *Saccharomyces cerevisiae* also expressing xylose reductase and xylitol dehydrogenase and its effect on fermentation of xylose and lignocellulosic hydrolysate. Appl Environ Microbiol. 2001 9 1;67(9):4249–55. 10.1128/AEM.67.9.4249-4255.2001 11526030 PMC93154

[15] Toivari MH, Aristidou A, Ruohonen L, Penttilä M. Conversion of xylose to ethanol by recombinant *Saccharomyces cerevisiae*: importance of xylulokinase (*XKS1*) and oxygen availability. Metab Eng. 2001 7 1;3(3):236–49. 10.1006/mben.2000.0191 11461146

[16] Jin Y-S, Jones S, Shi N-Q, Jeffries TW. Molecular cloning of *XYL3* (D-xylulokinase) from *Pichia stipitis* and characterization of its physiological function. Appl Environ Microbiol. 2002 3 1;68(3):1232–9. 10.1128/AEM.68.3.1232-1239.2002 11872473 PMC123745

[17] Jin Y-S, Ni H, Laplaza JM, Jeffries TW. Optimal growth and ethanol production from xylose by recombinant *Saccharomyces cerevisiae* require moderate D-xylulokinase activity. Appl Environ Microbiol. 2003 1 1;69(1):495–503. 10.1128/AEM.69.1.495-503.2003 12514033 PMC152454

[18] Kim SR, Ha S-J, Wei N, Oh EJ, Jin Y-S. Simultaneous co-fermentation of mixed sugars: a promising strategy for producing cellulosic ethanol. Trends Biotechnol. 2012 5 1;30(5):274–82. 10.1016/j.tibtech.2012.01.005 22356718

[19] Kim SR, Skerker JM, Kang W, Lesmana A, Wei N, Arkin AP, et al Rational and evolutionary engineering approaches uncover a small set of genetic changes efficient for rapid xylose fermentation in *Saccharomyces cerevisiae*. PLoS One. 2013 2 26;8(2):e57048 10.1371/journal.pone.0057048 23468911 PMC3582614

[20] Diao L, Liu Y, Qian F, Yang J, Jiang Y, Yang S. Construction of fast xylose-fermenting yeast based on industrial ethanol-producing diploid *Saccharomyces cerevisiae* by rational design and adaptive evolution. BMC Biotechnol. 2013 12 19;13(1):110. 24354503 10.1186/1472-6750-13-110 PMC3878346

[21] Parreiras LS, Breuer RJ, Avanasi Narasimhan R, Higbee AJ, La Reau A, Tremaine M, et al Engineering and two-stage evolution of a lignocellulosic hydrolysate-tolerant *Saccharomyces cerevisiae* strain for anaerobic fermentation of xylose from AFEX pretreated corn stover. PLoS One. 2014 9 15;9(9):e107499 10.1371/journal.pone.0107499 25222864 PMC4164640

[22] Sato TK, Tremaine M, Parreiras LS, Hebert AS, Myers KS, Higbee AJ, et al Directed evolution reveals unexpected epistatic interactions that alter metabolic regulation and enable anaerobic xylose use by *Saccharomyces cerevisiae*. PLOS Genet. 2016 10 14;12(10):e1006372 10.1371/journal.pgen.1006372 27741250 PMC5065143

[23] dos Santos LV, Carazzolle MF, Nagamatsu ST, Sampaio NMV, Almeida LD, Pirolla RAS, et al Unraveling the genetic basis of xylose consumption in engineered *Saccharomyces cerevisiae* strains. Sci Rep. 2016 12 21;6(1):38676. 28000736 10.1038/srep38676 PMC5175268

[24] Conrad M, Schothorst J, Kankipati HN, Van Zeebroeck G, Rubio-Texeira M, Thevelein JM. Nutrient sensing and signaling in the yeast *Saccharomyces cerevisiae*. FEMS Microbiol Rev. 2014 3 1;38(2):254–99. 10.1111/1574-6976.12065 24483210 PMC4238866

[25] Saito H, Posas F, Horecka J, DePinho RA, Sprague GF, Tyers M, et al Response to hyperosmotic stress. Genetics. 2012 10 1;192(2):289–318. 10.1534/genetics.112.140863 23028184 PMC3454867

[26] Remize F, Cambon B, Barnavon L, Dequin S. Glycerol formation during wine fermentation is mainly linked to Gpd1p and is only partially controlled by the HOG pathway. Yeast. 2003 11 1;20(15):1243–53. 10.1002/yea.1041 14618562

[27] Jiménez-Martí E, Zuzuarregui A, Gomar-Alba M, Gutiérrez D, Gil C, del Olmo M. Molecular response of S*accharomyces cerevisiae* wine and laboratory strains to high sugar stress conditions. Int J Food Microbiol. 2011 1 31;145(1):211–20. 10.1016/j.ijfoodmicro.2010.12.023 21247650

[28] Piao H, MacLean Freed J, Mayinger P. Metabolic activation of the HOG MAP kinase pathway by Snf1/AMPK regulates lipid signaling at the golgi. Traffic. 2012 11 1;13(11):1522–31. 10.1111/j.1600-0854.2012.01406.x 22882253 PMC3465495

[29] Tomás-Cobos L, Casadomé L, Mas G, Sanz P, Posas F. Expression of the *HXT1* low affinity glucose transporter requires the coordinated activities of the HOG and glucose signalling pathways. J Biol Chem. 2004 5 21;279(21):22010–9. 10.1074/jbc.M400609200 15014083

[30] Gomar-Alba M, Morcillo-Parra MÁ, Olmo M del. Response of yeast cells to high glucose involves molecular and physiological differences when compared to other osmostress conditions. FEMS Yeast Res. 2015 8 1;15(5):fov039 10.1093/femsyr/fov039 26048894

[31] Ho Y-H, Gasch AP. Exploiting the yeast stress-activated signaling network to inform on stress biology and disease signaling. Curr Genet. 2015 11 10;61(4):503–11. 10.1007/s00294-015-0491-0 25957506 PMC4800808

[32] Ask M, Bettiga M, Duraiswamy V, Olsson L. Pulsed addition of HMF and furfural to batch-grown xylose-utilizing S*accharomyces cerevisiae* results in different physiological responses in glucose and xylose consumption phase. Biotechnol Biofuels. 2013 12 16;6(1):181 10.1186/1754-6834-6-181 24341320 PMC3878631

[33] Piotrowski JS, Zhang Y, Bates DM, Keating DH, Sato TK, Ong IM, et al Death by a thousand cuts: the challenges and diverse landscape of lignocellulosic hydrolysate inhibitors. Front Microbiol. 2014 3 14;5:90 10.3389/fmicb.2014.00090 24672514 PMC3954026

[34] Almeida JRM, Runquist D, Sànchez Nogué V, Lidén G, Gorwa-Grauslund MF. Stress-related challenges in pentose fermentation to ethanol by the yeast *Saccharomyces cerevisiae*. Biotechnol J. 2011 3 1;6(3):286–99. 10.1002/biot.201000301 21305697

[35] Modig T, Lidén G, Taherzadeh MJ. Inhibition effects of furfural on alcohol dehydrogenase, aldehyde dehydrogenase and pyruvate dehydrogenase. Biochem J. 2002 5 1;363(Pt 3):769–76. 10.1042/0264-6021:3630769 11964178 PMC1222530

[36] Heer D, Sauer U. Identification of furfural as a key toxin in lignocellulosic hydrolysates and evolution of a tolerant yeast strain. Microb Biotechnol. 2008 11 1;1(6):497–506. 10.1111/j.1751-7915.2008.00050.x 21261870 PMC3815291

[37] Marchler G, Schüller C, Adam G, Ruis H. A *Saccharomyces cerevisiae* UAS element controlled by protein kinase A activates transcription in response to a variety of stress conditions. EMBO J. 1993 5 1;12(5):1997–2003. 8387917 10.1002/j.1460-2075.1993.tb05849.x PMC413422

[38] Stanhill A, Schick N, Engelberg D. The yeast ras/cyclic AMP pathway induces invasive growth by suppressing the cellular stress response. Mol Cell Biol. 1999 11 1;19(11):7529–38. 10.1128/mcb.19.11.7529 10523641 PMC84760

[39] Trott A, Shaner L, Morano KA. The molecular chaperone Sse1 and the growth control protein kinase Sch9 collaborate to regulate protein kinase A activity in *Saccharomyces cerevisiae*. Genetics. 2005 7;170(3):1009–21. 10.1534/genetics.105.043109 15879503 PMC1451167

[40] Park J-I, Collinson EJ, Grant CM, Dawes IW. Rom2p, the Rho1 GTP/GDP exchange factor of *Saccharomyces cerevisiae*, can mediate stress responses via the Ras-cAMP pathway. J Biol Chem. 2005 1 28;280(4):2529–35. 10.1074/jbc.M407900200 15545276

[41] Sasano Y, Watanabe D, Ukibe K, Inai T, Ohtsu I, Shimoi H, et al Overexpression of the yeast transcription activator Msn2 confers furfural resistance and increases the initial fermentation rate in ethanol production. J Biosci Bioeng. 2012 4 1;113(4):451–5. 10.1016/j.jbiosc.2011.11.017 22178024

[42] Wallace-Salinas V, Signori L, Li Y-Y, Ask M, Bettiga M, Porro D, et al Re-assessment of *YAP1* and *MCR1* contributions to inhibitor tolerance in robust engineered *Saccharomyces cerevisiae* fermenting undetoxified lignocellulosic hydrolysate. AMB Express. 2014 12 22;4(1):56. 25147754 10.1186/s13568-014-0056-5 PMC4105880

[43] Oh EJ, Wei N, Kwak S, Kim H, Jin Y-S. Overexpression of *RCK1* improves acetic acid tolerance in *Saccharomyces cerevisiae*. J Biotechnol. 2019 2 20;292:1–4. 10.1016/j.jbiotec.2018.12.013 30615911

[44] Almeida JRM, Bertilsson M, Hahn-Hägerdal B, Lidén G, Gorwa-Grauslund M-F. Carbon fluxes of xylose-consuming *Saccharomyces cerevisiae* strains are affected differently by NADH and NADPH usage in HMF reduction. Appl Microbiol Biotechnol. 2009 9 9;84(4):751–61. 10.1007/s00253-009-2053-1 19506862

[45] Zheng D-Q, Wu X-C, Tao X-L, Wang P-M, Li P, Chi X-Q, et al Screening and construction of *Saccharomyces cerevisiae* strains with improved multi-tolerance and bioethanol fermentation performance. Bioresour Technol. 2011 2 1;102(3):3020–7. 10.1016/j.biortech.2010.09.122 20980141

[46] Demeke MM, Dumortier F, Li Y, Broeckx T, Foulquié-Moreno MR, Thevelein JM. Combining inhibitor tolerance and D-xylose fermentation in industrial Saccharomyces cerevisiae for efficient lignocellulose-based bioethanol production. Biotechnol Biofuels. 2013 8 26;6(1):120 10.1186/1754-6834-6-120 23971950 PMC3765968

[47] Smith J, van Rensburg E, Görgens JF. Simultaneously improving xylose fermentation and tolerance to lignocellulosic inhibitors through evolutionary engineering of recombinant *Saccharomyces cerevisiae* harbouring xylose isomerase. BMC Biotechnol. 2014 May 15;14(1):41. 24884721 10.1186/1472-6750-14-41 PMC4026109

[48] Bellissimi E, van Dijken JP, Pronk JT, van Maris AJA. Effects of acetic acid on the kinetics of xylose fermentation by an engineered, xylose-isomerase-based *Saccharomyces cerevisiae* strain. FEMS Yeast Res. 2009 5 1;9(3):358–64. 10.1111/j.1567-1364.2009.00487.x 19416101

[49] Myers KS, Riley NM, MacGilvray ME, Sato TK, McGee M, Heilberger J, et al Rewired cellular signaling coordinates sugar and hypoxic responses for anaerobic xylose fermentation in yeast. PLOS Genet. 2019 3 11;15(3):e1008037 10.1371/journal.pgen.1008037 30856163 PMC6428351

[50] Ho CH, Magtanong L, Barker SL, Gresham D, Nishimura S, Natarajan P, et al A molecular barcoded yeast ORF library enables mode-of-action analysis of bioactive compounds. Nat Biotechnol. 2009 4 6;27(4):369–77. 10.1038/nbt.1534 19349972 PMC3856559

[51] Sherman F. Getting started with yeast. Methods Enzymol. 2002 1 1;350:3–41. 12073320 10.1016/s0076-6879(02)50954-x

[52] Chundawat SPS, Vismeh R, Sharma LN, Humpula JF, da Costa Sousa L, Chambliss CK, et al Multifaceted characterization of cell wall decomposition products formed during ammonia fiber expansion (AFEX) and dilute acid based pretreatments. Bioresour Technol. 2010 11 1;101(21):8429–38. 10.1016/j.biortech.2010.06.027 20598525

[53] Sardi M, Rovinskiy N, Zhang Y, Gasch AP. Leveraging genetic-background effects in *Saccharomyces cerevisiae* to improve lignocellulosic hydrolysate tolerance. Appl Environ Microbiol. 2016 10 1;82(19):5838–49. 10.1128/AEM.01603-16 27451446 PMC5038035

[54] Balan V, Bals B, Chundawat SPS, Marshall D, Dale BE. Lignocellulosic biomass pretreatment using AFEX In Humana Press, Totowa, NJ; 2009 p. 61–77. 10.1007/978-1-60761-214-8_5 19768616

[55] Wenger CD, Phanstiel DH, Lee MV, Bailey DJ, Coon JJ. COMPASS: A suite of pre- and post-search proteomics software tools for OMSSA. Proteomics. 2011 3 1;11(6):1064–74. 10.1002/pmic.201000616 21298793 PMC3049964

[56] Geer LY, Markey SP, Kowalak JA, Wagner L, Xu M, Maynard DM, Yang X, Shi W, Bryant SH. Open mass spectrometry search algorithm. J Proteome Res. 2004 10 11;3(5):958–64. 10.1021/pr0499491 15473683

[57] Merrill AE, Hebert AS, MacGilvray ME, Rose CM, Bailey DJ, Bradley JC, et al NeuCode labels for relative protein quantification. Mol Cell Proteomics. 2014 9 17;13(9):2503–12. 10.1074/mcp.M114.040287 24938287 PMC4159665

[58] Thevelein JM, de Winde JH. Novel sensing mechanisms and targets for the cAMP-protein kinase A pathway in the yeast *Saccharomyces cerevisiae*. Mol Microbiol. 1999 9 1;33(5):904–18. 10476026 10.1046/j.1365-2958.1999.01538.x

[59] Neuman-Silberberg FS, Bhattacharya S, Broach JR. Nutrient availability and the RAS/cyclic AMP pathway both induce expression of ribosomal protein genes in *Saccharomyces cerevisiae* but by different mechanisms. Mol Cell Biol. 1995 6 1;15(6):3187–96. 10.1128/mcb.15.6.3187 7760815 PMC230551

[60] Klein C, Struhl K. Protein kinase A mediates growth-regulated expression of yeast ribosomal protein genes by modulating *RAP1* transcriptional activity. Mol Cell Biol. 1994 3 1;14(3):1920–8. 10.1128/mcb.14.3.1920 8114723 PMC358550

[61] Rittenhouse J, Moberly L, Marcus F. Phosphorylation in vivo of yeast (*Saccharomyces cerevisiae*) fructose-1,6-bisphosphatase at the cyclic AMP-dependent site. J Biol Chem. 1987 7 25;262(21):10114–9. 3038868

[62] Portela P, Howell S, Moreno S, Rossi S. *In vivo* and *in vitro* phosphorylation of two isoforms of yeast pyruvate kinase by protein kinase A. J Biol Chem. 2002 8 23;277(34):30477–87. 10.1074/jbc.M201094200 12063246

[63] Dihazi H, Kessler R, Eschrich K. Glucose-induced stimulation of the Ras-cAMP pathway in yeast leads to multiple phosphorylations and activation of 6-phosphofructo-2-kinase. Biochemistry. 2003 5 27;42(20):6275–82. 10.1021/bi034167r 12755632

[64] François J, Parrou JL. Reserve carbohydrates metabolism in the yeast *Saccharomyces cerevisiae*. FEMS Microbiol Rev. 2001 1 1;25(1):125–45. 10.1111/j.1574-6976.2001.tb00574.x 11152943

[65] Enjalbert B, Parrou JL, Teste MA, François J. Combinatorial control by the protein kinases PKA, PHO85 and SNF1 of transcriptional induction of the *Saccharomyces cerevisiae GSY2* gene at the diauxic shift. Mol Genet Genomics. 2004 7 22;271(6):697–708. 10.1007/s00438-004-1014-8 15221454

[66] Hirimburegama K, Durnez P, Keleman J, Oris E, Vergauwen R, Mergelsberg H, et al Nutrient-induced activation of trehalase in nutrient-starved cells of the yeast *Saccharomyces cerevisiae*: cAMP is not involved as second messenger. J Gen Microbiol. 1992 10 1;138(10):2035–43. 10.1099/00221287-138-10-2035 1336029

[67] Durnez P, Pernambuco MB, Oris E, Argüelles J-C, Mergelsberg H, Thevelein JM. Activation of trehalase during growth induction by nitrogen sources in the yeast *Saccharomyces cerevisiae* depends on the free catalytic subunits of camp-dependent protein kinase, but not on functional ras proteins. Yeast. 1994 8 1;10(8):1049–64. 10.1002/yea.320100807 7992505

[68] Schepers W, Van Zeebroeck G, Pinkse M, Verhaert P, Thevelein JM. *In vivo* phosphorylation of Ser21 and Ser83 during nutrient-induced activation of the yeast protein kinase A (PKA) target trehalase. J Biol Chem. 2012 12 28;287(53):44130–42. 10.1074/jbc.M112.421503 23155055 PMC3531729

[69] Ewald JC, Kuehne A, Zamboni N, Skotheim JM. The yeast cyclin-dependent kinase routes carbon fluxes to fuel cell cycle progression. Mol Cell. 2016 5 19;62(4):532–45. 10.1016/j.molcel.2016.02.017 27203178 PMC4875507

[70] Vallejo MC, Mayinger P. Delayed turnover of unphosphorylated Ssk1 during carbon stress activates the yeast Hog1 MAP kinase pathway. PLoS One. 2015 9 4;10(9):e0137199 10.1371/journal.pone.0137199 26340004 PMC4560374

[71] Dihazi H, Kessler R, Eschrich K. High osmolarity glycerol (HOG) pathway-induced phosphorylation and activation of 6-phosphofructo-2-kinase are essential for glycerol accumulation and yeast cell proliferation under hyperosmotic stress. J Biol Chem. 2004 6 4;279(23):23961–8. 10.1074/jbc.M312974200 15037628

[72] MacGilvray ME, Shishkova E, Chasman D, Place M, Gitter A, Coon JJ, et al Network inference reveals novel connections in pathways regulating growth and defense in the yeast salt response. PLOS Comput Biol. 2018 5 8;13(5):e1006088 10.1371/journal.pcbi.1006088 29738528 PMC5940180

[73] Albertyn J, Hohmann S, Thevelein JM, Prior BA. *GPD1*, which encodes glycerol-3-phosphate dehydrogenase, is essential for growth under osmotic stress in *Saccharomyces cerevisiae*, and its expression is regulated by the high-osmolarity glycerol response pathway. Mol Cell Biol. 1994 6 1;14(6):4135–44. 10.1128/mcb.14.6.4135 8196651 PMC358779

[74] Brewster JL, Gustin MC. Positioning of cell growth and division after osmotic stress requires a MAP kinase pathway. Yeast. 1994 4 1;10(4):425–39. 10.1002/yea.320100402 7941729

[75] Capaldi AP, Kaplan T, Liu Y, Habib N, Regev A, Friedman N, et al Structure and function of a transcriptional network activated by the MAPK Hog1. Nat Genet. 2008 11 19;40(11):1300–6. 10.1038/ng.235 18931682 PMC2825711

[76] Kühn C, Petelenz E, Nordlander B, Schaber J, Hohmann S, Klipp E. Exploring the impact of osmoadaptation on glycolysis using time-varying response-coefficients. In Genome Informatics 2008: Genome Informatics Series Vol. 20 2008 (pp. 77–90). 19425124

[77] Petelenz-Kurdziel E, Kuehn C, Nordlander B, Klein D, Hong K-K, Jacobson T, et al Quantitative analysis of glycerol accumulation, glycolysis and growth under hyper osmotic stress. PLoS Comput Biol. 2013 6 6;9(6):e1003084 10.1371/journal.pcbi.1003084 23762021 PMC3677637

[78] Babazadeh R, Furukawa T, Hohmann S, Furukawa K. Rewiring yeast osmostress signalling through the MAPK network reveals essential and non-essential roles of Hog1 in osmoadaptation. Sci Rep. 2015 5 15;4(1):4697. 10.1038/srep04697 PMC3986706 24732094

[79] Pisithkul T, Jacobson TB, O’Brien TJ, Stevenson DM, Amador-Noguez D. Phenolic amides are potent inhibitors of de novo nucleotide biosynthesis. Appl Environ Microbiol. 2015 9 1;81(17):5761–72. 10.1128/AEM.01324-15 26070680 PMC4551265

[80] Jayakody LN, Turner TL, Yun EJ, Kong II, Liu J-J, Jin Y-S. Expression of Gre2p improves tolerance of engineered xylose-fermenting *Saccharomyces cerevisiae* to glycolaldehyde under xylose metabolism. Appl Microbiol Biotechnol. 2018 9 19;102(18):8121–33. 10.1007/s00253-018-9216-x 30027490

[81] Pampulha ME, Loureiro-Dias MC. Activity of glycolytic enzymes of *Saccharomyces cerevisiae* in the presence of acetic acid. Appl Microbiol Biotechnol. 1990 12;34(3):375–80.

[82] Zhang G-C, Kong II, Wei N, Peng D, Turner TL, Sung BH, et al Optimization of an acetate reduction pathway for producing cellulosic ethanol by engineered yeast. Biotechnol Bioeng. 2016 12 1;113(12):2587–96. 10.1002/bit.26021 27240865

[83] Wei N, Quarterman J, Kim SR, Cate JHD, Jin Y-S. Enhanced biofuel production through coupled acetic acid and xylose consumption by engineered yeast. Nat Commun. 2013 12 8;4(1):2580. 24105024 10.1038/ncomms3580

[84] Colombo S, Ronchetti D, Thevelein JM, Winderickx J, Martegani E. Activation state of the Ras2 protein and glucose-induced signaling in *Saccharomyces cerevisiae*. J Biol Chem. 2004 11 5;279(45):46715–22. 10.1074/jbc.M405136200 15339905

[85] Vandamme J, Castermans D, Thevelein JM. Molecular mechanisms of feedback inhibition of protein kinase A on intracellular cAMP accumulation. Cell Signal. 2012 8 1;24(8):1610–8. 10.1016/j.cellsig.2012.04.001 22522182

[86] Peeters T, Louwet W, Geladé R, Nauwelaers D, Thevelein JM, Versele M. Kelch-repeat proteins interacting with the Galpha protein Gpa2 bypass adenylate cyclase for direct regulation of protein kinase A in yeast. Proc Natl Acad Sci U S A. 2006 8 29;103(35):13034–9. 10.1073/pnas.0509644103 16924114 PMC1559748

[87] Werner-Washburne M, Brown D, Braun E. Bcy1, the regulatory subunit of cAMP-dependent protein kinase in yeast, is differentially modified in response to the physiological status of the cell. J Biol Chem. 1991 10 15;266(29):19704–9. 1655793

[88] Griffioen G, Anghileri P, Imre E, Baroni MD, Ruis H. Nutritional control of nucleocytoplasmic localization of cAMP-dependent protein kinase catalytic and regulatory subunits in *Saccharomyces cerevisiae*. J Biol Chem. 2000 1 14;275(2):1449–56. 10.1074/jbc.275.2.1449 10625697

[89] Griffioen G, Branduardi P, Ballarini A, Anghileri P, Norbeck J, Baroni MD, et al Nucleocytoplasmic distribution of budding yeast protein kinase A regulatory subunit Bcy1 requires Zds1 and is regulated by Yak1-dependent phosphorylation of its targeting domain. Mol Cell Biol. 2001 1 15;21(2):511–23. 10.1128/MCB.21.2.511-523.2001 11134339 PMC86612

[90] Griffioen G, Swinnen S, Thevelein JM. Feedback inhibition on cell wall integrity signaling by Zds1 involves Gsk3 phosphorylation of a cAMP-dependent protein kinase regulatory subunit. J Biol Chem. 2003 6 27;278(26):23460–71. 10.1074/jbc.M210691200 12704202

[91] Zhang A, Gao W. Mechanisms of protein kinase Sch9 regulating Bcy1 in *Saccharomyces cerevisiae*. FEMS Microbiol Lett. 2012 6 1;331(1):10–6. 10.1111/j.1574-6968.2012.02552.x 22428880

[92] Smith FD, Esseltine JL, Nygren PJ, Veesler D, Byrne DP, Vonderach M, et al Local protein kinase A action proceeds through intact holoenzymes. Science. 2017 6 23;356(6344):1288–93. 10.1126/science.aaj1669 28642438 PMC5693252

[93] Vigil D, Blumenthal DK, Brown S, Taylor SS, Trewhella J. Differential effects of substrate on type I and type II PKA holoenzyme dissociation. Biochemistry. 2004 5 18;43(19):5629–36. 10.1021/bi0499157 15134437

