# Supplementary figures and images for: PKA and HOG signaling contribute separable roles to anaerobic xylose fermentation in yeast engineered for biofuel production
Source: PLoS One. 2019 May 21;14(5):e0212389. doi: 10.1371/journal.pone.0212389 (PMC6528989; doi:10.1371/journal.pone.0212389)

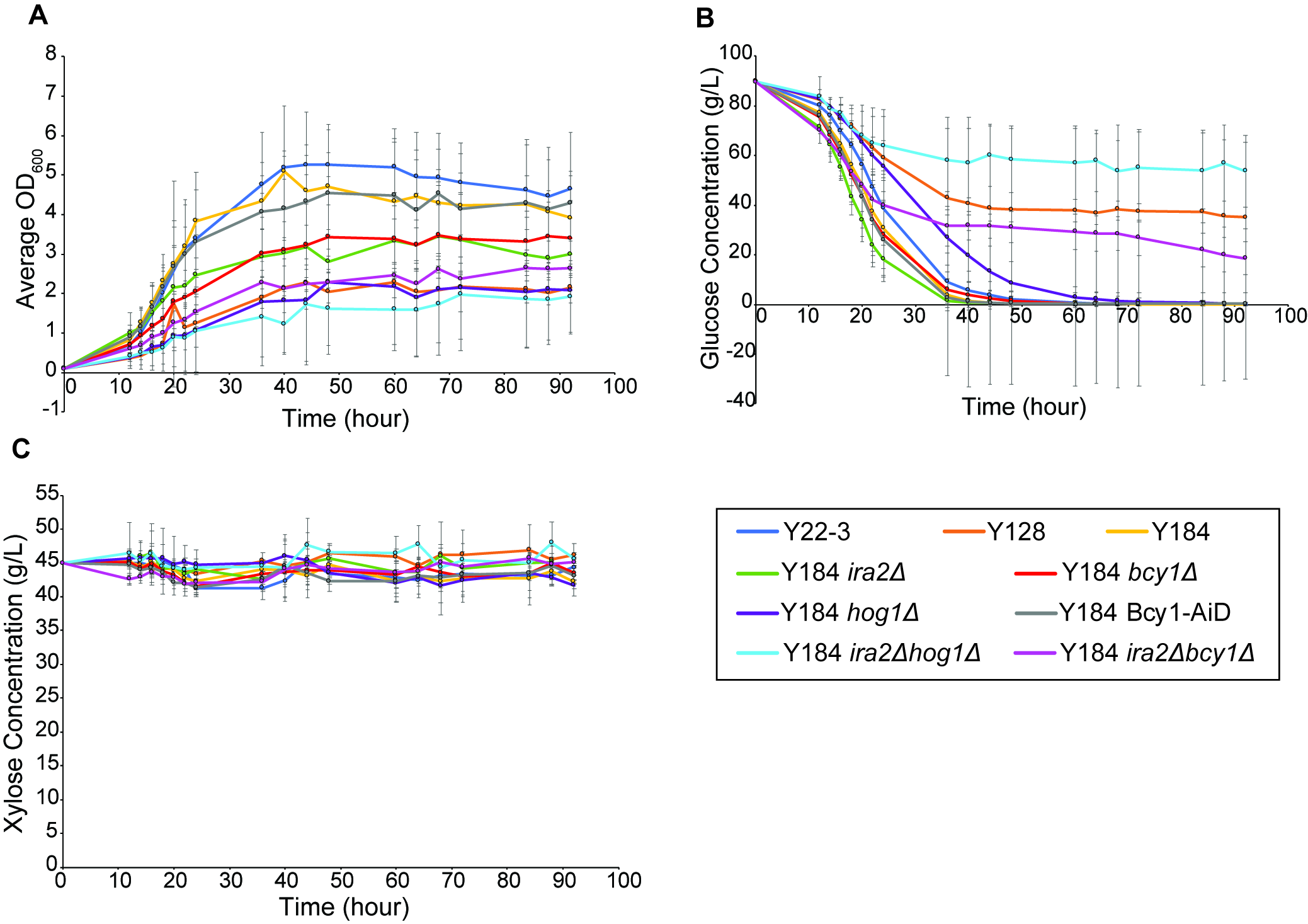

Supplement: S1 Fig — Batch cultures were grown in 9% ACSH anaerobically for 92 hours. Data represent average and standard deviation from three biological replicates. A. OD600 measurements over time. Glucose (B.) and xylose (C.) concentration in the media over time. (TIF) [file pone.0212389.s001.tif]

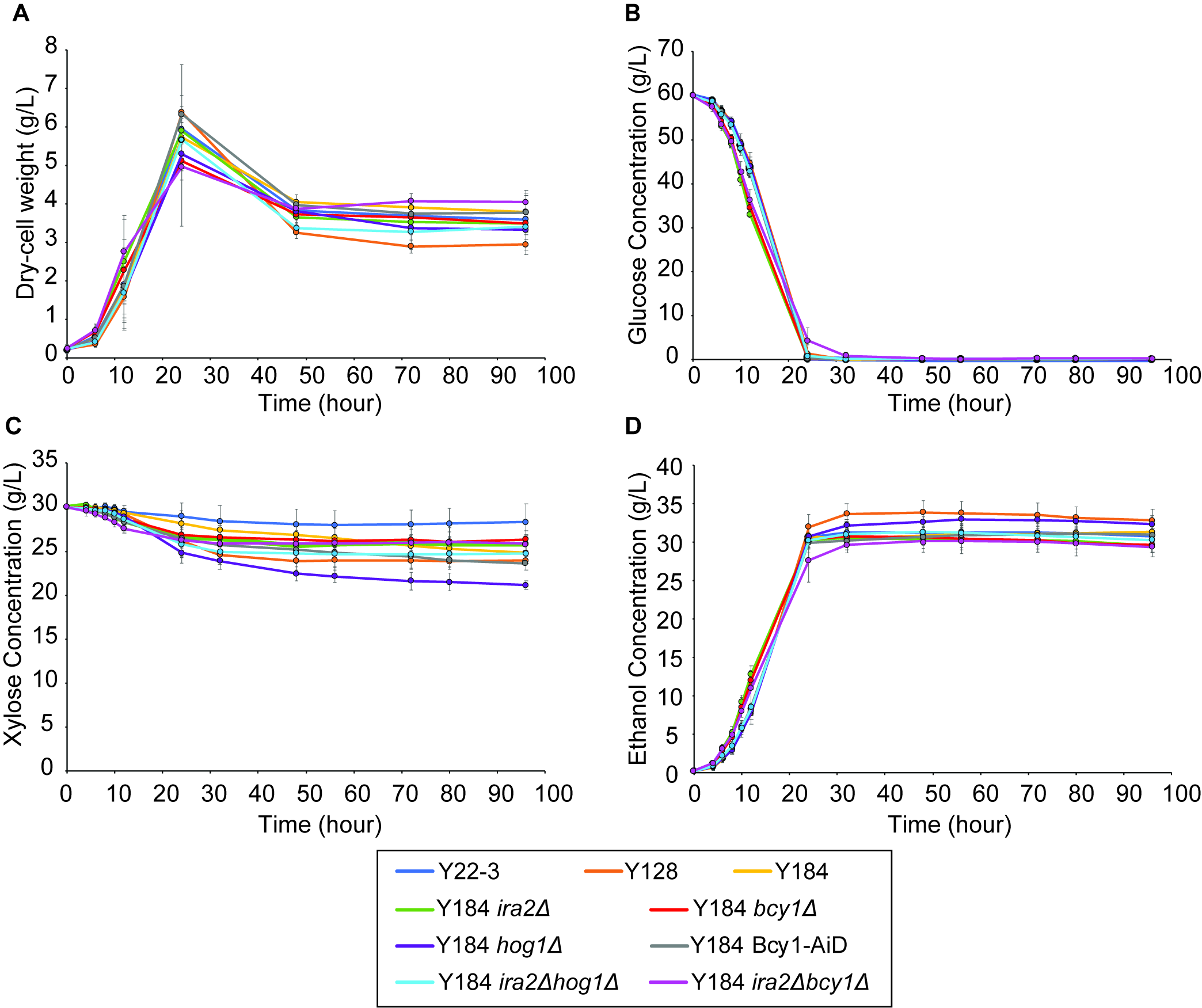

Supplement: S2 Fig — As described in S1 Fig except for anaerobic 6% ACSH growth, measuring dry-cell weight (A.), and glucose (B.), xylose (C.), and ethanol (D.) media concentration over time. (TIF) [file pone.0212389.s002.tif]

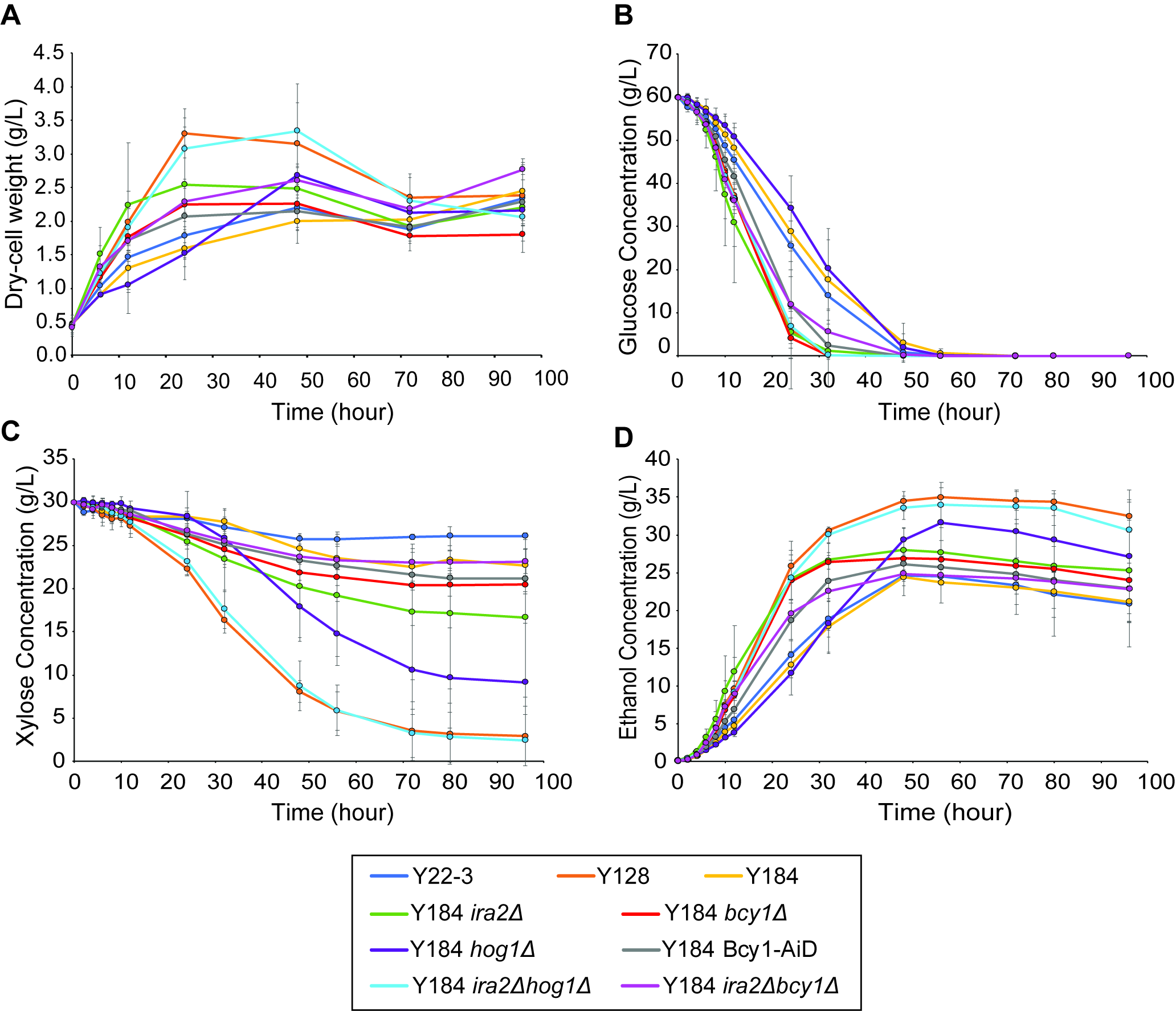

Supplement: S3 Fig — As described in S1 Fig except for anaerobic YPDX 6%/3% growth, measuring dry-cell weight (A.), and glucose (B.), xylose (C.), and ethanol (D.) media concentration over time. (TIF) [file pone.0212389.s003.tif]

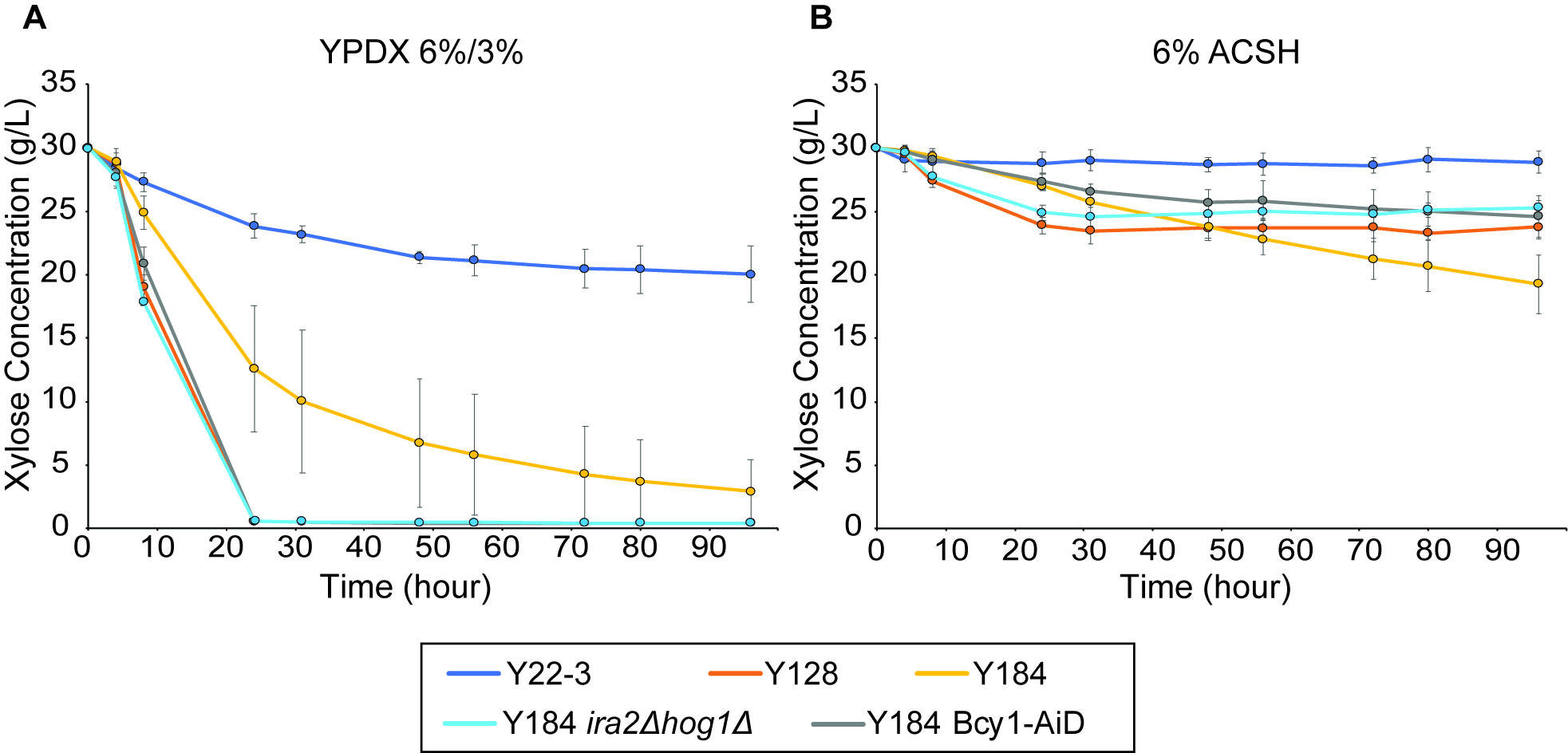

Supplement: S4 Fig — Batch cultures were grown anaerobically for 96 hours in YPDX 6%/3% (A.) or 6% ACSH (B.). Cultures were started at an OD600 of 3. Data represent average and standard deviation of three biological replicates. Comparing Panel A to Fig 3C shows that the Y184 Bcy1-AiD strain ferments xylose when the culture is inoculated at a higher starting OD but not when inoculated at a lower cell density. (TIF) [file pone.0212389.s004.tif]
